# Insights into the Structures, Inhibitors, and Improvement Strategies of Glucose Oxidase

**DOI:** 10.3390/ijms23179841

**Published:** 2022-08-30

**Authors:** Fan Wang, Xiaona Chen, Yonggang Wang, Xing Li, Minglai Wan, Ge Zhang, Feifan Leng, Haibo Zhang

**Affiliations:** 1 Qingdao Institute of Bioenergy and Bioprocess Technology, Chinese Academy of Sciences, Qingdao 266101, China; 2 School of Life Science and Engineering, Lanzhou University of Technology, Lanzhou 730050, China

**Keywords:** glucose oxidase, structures, inhibitors, yield improvement

## Abstract

Glucose oxidase, which uses molecular oxygen as an electron acceptor to specifically catalyze the conversion of β-d-glucose to gluconic acid and hydrogen peroxide (H_2_O_2_), has been considered an important enzyme in increasing environmental sustainability and food security. However, achieving the high yield, low price and high activity required for commercial viability remains challenging. In this review, we first present a brief introduction, looking at the sources, characteristics, catalytic process, and applications of glucose oxidase. Then, the predictive structures of glucose oxidase from two different sources are comparatively discussed. We summarize the inhibitors of glucose oxidase. Finally, we highlight how the production of glucose oxidase can be improved by optimizing the culture conditions and microbial metabolic engineering.

## 1. Introduction

**Figure 1:**
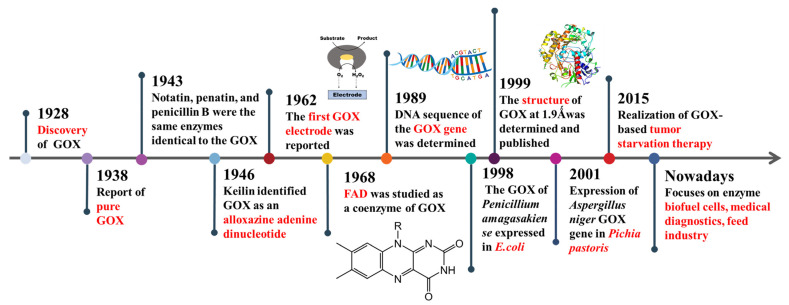
The development process of glucose oxidase.

Glucose oxidase (GOX, β-d-glucose: oxygen 1-oxidoreductase, EC 1.1.3.4) is a kind of dimeric protease with flavin adenine dinucleotide (FAD), belonging to the glucose/methanol/choline oxidoreductase family [1]. It is a nearly white or light-yellow powder, easily soluble in water, almost insoluble in organic solvents, and an aerobic dehydrogenase with an optimal pH of 3.5–6.5 and optimal temperature of 30–60 °C [2]. In previous work, GOX showed the highest activity against β-d-glucose with a relative enzyme activity of 100%, while trehalose, d-galactose, melibiose, and raffinose show the values of 5.2%, 4.6%, 3.95%, 0.6%, respectively [3]. The development process of GOX is shown in Figure 1 and the source, structure, catalytic mechanism, and characteristics of GOX were introduced before 2000.

GOX is naturally produced by animals, plants, and microorganisms. Although animals and plants are important GOX producers, their low yield and non-economical extraction and separation process limit their use in manufacturing. Thus, GOX is produced by microorganisms in the long term, mostly *Penicillium* and *Aspergillus*, at an industrial scale. The main advantage of using microorganisms depends on their ability to quickly convert carbon sources into GOX with high productivity. Few wild bacterial strains for that produce GOX have been reported to date [4]. Table 1 lists details of the strains for GOX production, and its molecular weight and characterization.

**Figure 2:**
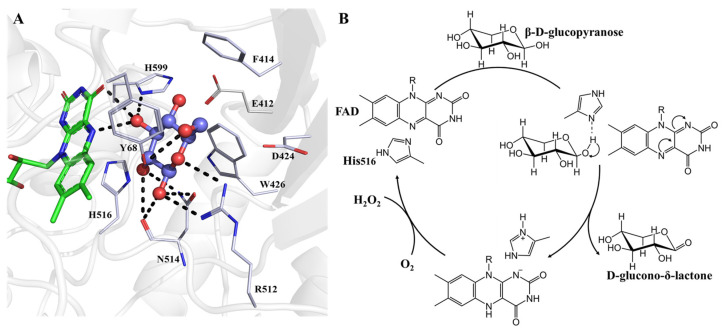
(**A**) The active site and amino acid residues of the oxidized form of the GOX from *Aspergillus niger* (PDB:1CF3) in the present of β-d-glucose. (**B**) Reaction scheme of glucose oxidation catalyzed by glucose oxidase.

In the catalytic process, GOX uses molecular oxygen as an electron acceptor to specifically catalyze β-d-glucose to d-glucono-δ-lactone and hydrogen peroxide (H_2_O_2_), using the FAD coenzyme as an electron carrier [24,25,26]. d-glucono-δ-lactone is further hydrolyzed into gluconic acid, while the generated H_2_O_2_ is broken down to O_2_ and H_2_O by catalase [1]. This process can also be expressed by two half-reactions. In the reductive half reaction, FAD is reduced to FADH_2_ by accepting electrons, and GOX catalyzes β-d-glucose to produce δ-gluconolactone, which is then non-enzymically hydrolyzed to gluconic acid. In the oxidation half-reaction, FADH_2_ is oxidized to FAD, and molecular oxygen accepts electrons and protons to form H_2_O_2_ [27,28]. The essential catalytic sites of GOX from *Aspergillus niger* are shown in Figure 2A and the entire catalytic process is shown in Figure 2B. The catalytic action of GOX is dependent on the FAD cofactor, which helps with electron transfer in the catalytic process. The FAD active site is provided by a deep pocket, which is located at the bottom of the pocket and has several main amino acid side chains nearby, including Glu412, His516, and His559 [29]. These three amino acids are essential for the catalytic process. His559 is strongly bound to Glu412 and a water molecule in front of FAD with a hydrogen bond [27]. An interaction map of these three amino acids is shown by Bauer. et al. [4]. His516/520 and His559/563 are potential proton receptors in *Aspergillus niger* and *Penicillium amagasakiense* GOX, respectively [28].

GOX is considered to be an important enzyme that can be applied in bioelectrochemistry, food, pharmaceutical, medical diagnostic, and feed field. The use of GOX in biosensors and biofuel cells has previously been fully discussed in terms of clinical and personal use [30], glucose monitoring in diabetic patients [31], and reengineering GOX to better serve glucose sensors and biofuel cells [32]. GOX is a natural, non-toxic additive in the food industry for food spoilage microorganisms (*Staphylococcus aureus*, *Salmonella infantis*, *Clostridium perfringens*, *Bacillus cereus* and *Listeria monocytogenes*) prevention [7], chemical additives (such as, potassium bromate) replacement [33], browning and deterioration retardation [34,35,36]. Cancer-related applications of GOX mainly focus on diagnosis and treatment, which were summarized in detail by Fu et al. [37] and Wang et al. [38]. A recent overview systematically addressed GOX applications in the feed field and the mechanism of its promoting animal growth [39]. Hence, this review does not systematically discuss the application of GOX.

In this review, the function, production, and properties of GOX summarized in previous reviews are briefly introduced [1,4,24,40]. The predictive structures of GOX from *Heliothis viriplaca* and *Citrobacter freundii* were described and compared with a known structure of *Aspergillus niger* GOX. The inhibitors of GOX are introduced through an extensive assessment of the literature. Related strategies for enhancing GOX production and activity are highlighted in the discussion.

## 2. The Structure of Glucose Oxidase

**Figure 3:**
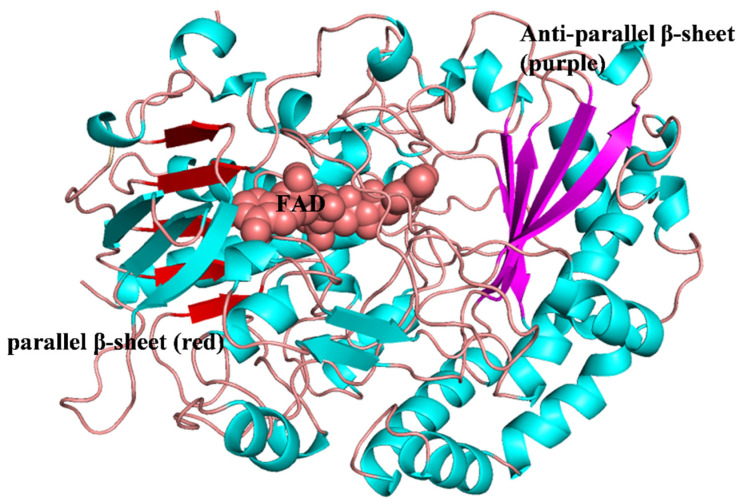
The structure of glucose oxidase from *Penicillium amagasakiense*.

The GOX from structures derived *Penicillium* and *Aspergillus* have been well-studied. They consist of two identical subunits, and each monomer has two distinct domains: one is mainly a β-sheet, and is tightly but not covalently bound with FAD; the other is supported by four α-helices and an antiparallel β-sheet, and binds to the substrates (Figure 3) [41]. FAD binding to GOX is an essential step in its function. The current research on the structures and properties of GOX mainly look at *Aspergillus niger* and *Penicillium amagasakiense*. The molecular weight mainly depends on the degree of glycosylation, and ranges from approximately 130 to 175 kDa [25]. GOX from *Aspergillus niger* is a homodimeric flavoprotein containing N- and O-glycosylation [42]. The GOX of *Penicillium amagasakiense* is glycosylated with a mannose rich carbohydrate, and the removal of carbohydrates from the enzyme shows no effect on the structure, activity, or stability of the enzyme [25,43].

The crystal structure of GOX from *Aspergillus niger* and *Penicillium amagasakiense* has been confirmed with 1.9 Å resolution (PDB 1CF3) and 1.8 Å resolution (PDB 1GPE), respectively [44]. Other examples can be found in PDB (1GAL, 3QVP, 3QVR, 5NIT, 5NIW). The structure of GOX from *Aspergillus niger* and *Penicillium amagasakiense* has been analyzed in detail, but the structures and analysis of GOX from other strains are still lacking. For studying GOX from different strains, ProtParam (https://web.expasy.org/protparam/ (accessed on 16 February 2022)) can be used to make predictions about some of their properties, and the Phyre2 web can be employed to predict their protein structures [45]. Compared with the well-known structures of *Aspergillus niger*, the GOX structures derived from fungi do not have so many differences, while the structures from insects and bacteria are quite different. Here, *Aspergillus niger* sequences (PDB 1CF3) and other sequences were aligned using ClustalW (https://www.genome.jp/tools-bin/clustalw (accessed on 15 April 2022)). The results showed that the GOX protein structures from *Aspergillus* (GenBank: RMZ47306.1, AHC55209.1), *Penicillium* (GenBank: AFA42947.1, ABM63225.1) and *Talaromyces* (GenBank: AAB09442.1, CAE47418.1) have a high similarity (60–80%), while the other species have a low similarity (15–30%). Although the amino-acid sequence alignment and structures of GOX from some fungi and insects have been described and discussed by Bauer et al. [4], GOX from *Citrobacter freundii* and *Heliothis viriplaca* has not been fully overviewed. *Citrobacter freundii* is the only bacterial strain that has been reported in Chinese regarding GOX production, and the sequence has been submitted to the NCBI database [46].

**Figure 4:**
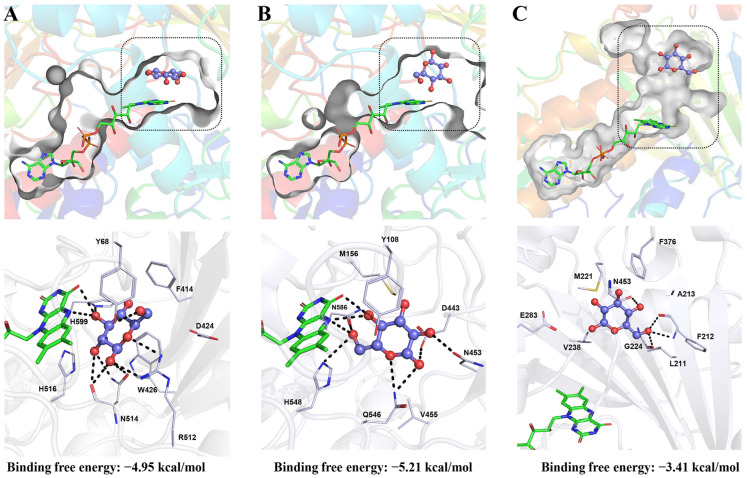
The crystal structure and substrate binding pocket of GOD from *Aspergillus niger* (**A**) with *Heliothis viriplaca* (**B**) and *Citrobacter freundii* (**C**) with the binding free energy. The predictive substrate binding pockets are shown in dotted boxes.

Figure 4 presents the predictive structures of GOX derived from *Citrobacter freundii* (GenBank: QGP74835.1) and *Heliothis viriplaca* (GenBank: AMR44226.1) with a 12% and 20% similarity in their amino-acid sequence, respectively, compared with the well-known structure from *Aspergillus niger.* Their structures and substrate binding pockets are greatly varied. Despite this, it has been reported that they showed the same feature when specifically catalyzing β-d-glucose with high or low efficiency. As shown in Figure 4A,B, the GOX from *Heliothis viriplaca* is very close to the expected one, with an analogous binding pocket and an approximative binding free energy; while their residues remain independent. It is believed that residues in loops, either remote or near the active site, can affect substrate recognition and catalysis by conformational changes and reshaping the binding pocket [47]. This could be the reason that the GOX from *Aspergillus niger* and *Heliothis viriplaca* performed differently. Meanwhile, the predictive structures of GOX from *Citrobacter freundii* indicates more obvious differences, not just from the active site but also from the binding pocket (Figure 4A,C). The binding free energy of GOX from *Citrobacter freundii* (−3.41 kcal/mol) indicates that it has a lower affinity to the surrounding amino-acid compared with the other two GOX. This may partially give explanatory notes to the fact that few GOX from bacteria has been in-depth investigated.

These results pinpoint the discrepancies between GOX from different sources, especially from animals and bacteria. The lack of a precise crystal structure and knowledge of the molecular mechanism of these GOXs further impedes the full comprehension of different types of GOX. Given the ongoing improvements in accurate protein structure prediction (such as AlphaFold) and structure analysis [48], we can now envision a future in which GOX are understood in depth, and find wide applications. In addition, further investigation to uncover the crystal structures of GOX complexed with inhibitors for improving its performance could provide insight into how the inhibitors affect the structures, substrate recognition, and function of GOX.

## 3. Inhibitors of Glucose Oxidase

Inhibitors can cause a reduction in enzyme activity or enzyme inactivation. Enzyme inhibitors are selective and can be an impetus to elucidating enzymes’ mechanism of action and metabolic pathways. Only by understanding the inhibitor’s mode of action can we come up with a solution to provide GOX with better activity and stability in the industry. GOX activity can be inhibited by metal ions (Ag^+^, Co^2+^, Hg^2+^) [49] and chemical reagents (H_2_O_2_, hydroxylamine, hydrazine, phenylhydrazone, dimedone, sodium bisulfate, guanidine hydrochloride, urea, sodium dodecyl sulfate) [40,50]. Ag^+^ can inhibit the oxidation of GOX by competing with molecular oxygen and combining with amino acid residues in or near the FAD moiety to reduce enzyme activity [51]. Hg^2+^ dilutes enzyme activity by inhibiting the oxidation of the reduced enzyme and weakening the binding of D-glucose to the enzyme with steric hindrance [51]. The research showed that Ag^+^, Co^2+^, Hg^2+^ at 1 mM concentration exhibited obvious inhibitions of GOX activity [49].

H_2_O_2_ is a competitive inhibitor of GOX [52], and can oxidize methionine (Met), a sulfur-containing amino acid in GOX to methionine sulfoxide and lead to the inactivation of GOX [50]. A total of 40% of GOX activity was lost in the presence of 200 mM of H_2_O_2_ [53]. In addition, the guanidine could enter the active pocket of GOX and interact with FAD resulting in a significant structural change between FAD and the surrounding amino acid residues, which mainly induces the conversion of α-helix to β-sheet structures leading to a reduction in the catalytic activity of GOX [54]. Thus, these inhibitors demonstrate high activity inhibition and structural transformation, potentially as a reference to avoid GOX inactivation and improve GOX yields.

## 4. Strategies for Improving Glucose Oxidase Yields

### 4.1. Optimization of Culture Conditions

In the of GOX production using microorganisms, high yields are aided by the provision of an appropriate culture medium and conditions. The components in the medium play important roles in the growth of microorganisms and biosynthesis of biochemicals. The carbon sources are responsible for the growth and metabolism of microorganisms, and provide cell’s carbon framework and provide the energy required for cell life activities. Meanwhile, the nitrogen source contains proteins and nucleic acids for living organisms. The study of different carbon and nitrogen sources that affected the yield and activity of GOX proposed that high levels of GOX were obtained using molasses as carbon source and peptone as nitrogen source [55]. A study stated that cultivation with only glucose and lactose helped to produce high levels of GOX compared with other sugars (fructose, sucrose, mannitol) [56]. Another example is the calcium carbonate that is essential in the production of GOX, which can have a significant impact, improving its activity [57].

In addition, the efficiency of microbial GOX production is usually dependent on temperature, pH, and dissolved oxygen. Most strains used for GOX production display an optimum temperature of 25–37 °C. The pH mainly affects the structure of bacterial cell membranes, the absorption and metabolism of nutrients, the activity of enzymes, and the direction of the metabolism. As reported in the literature, the optimum pH value for GOX production strains was in the range of 6.0–7.0 [25]. Dissolved oxygen also influences the organisms. Experimental evidence showed an increase in agitator speed corresponding to a higher concentration of dissolved oxygen, resulting in a higher growth rate and an increased production of GOX [58]. In addition, it is reported that the addition of hydrocarbons (n-dodecane, n-hexadecane) had a positive effect on the formation of intracellular and extracellular GOX [59]. These examples provide enough information for culturing a GOX-producing strain.

### 4.2. Mutation Technology

Mutagenesis is a primary and efficient way of engineering strains to change their performance, usually followed by a special environmental conditions screening for the desired mutations [60]. Common approaches to random mutagenesis include radiation mutagenesis (such as ultraviolet irradiation, laser and gamma irradiation) [61], chemical mutagenesis (for example, N-methyl-N-nitro-N-nitrosoguanidine or diethyl sulfate) [62], the protoplast fusion technique [63]. Physical mutations have the advantage of having a high frequency of multiple types of variants and the disadvantage of being very disruptive to chromosome structure [64]. The experimental results showed that mutagenesis of *Aspergillus niger* by UV irradiation resulted in a 1.57-fold and 1.98-fold increase in the in vitro and in vivo enzyme activities of the obtained mutant glucose oxidase, respectively [60]. Chemical mutagenesis typically induces point mutations, so these alleles often reveal previously unrecognized functions of known proteins, but the chemicals used are often toxic [65]. A study [62] revealed that N-methyl-N-nitro-N-nitrosoguanidine (MNNG) and ethidium bromide caused the wild *Aspergillus niger* to change, and the enzyme activity of the mutants increased by 282% and 202%, respectively. It was believed that the random mutagenesis of *Aspergillus tubingensis* using nitric acid and UV irradiation can screen for some mutants, leading to a significant yield improvement [66]. Meanwhile, Khattab et al. [63] selected mutants of *Aspergillus niger* and used them for protoplast fusion in intraspecific hybridization, resulting in fusants with higher activity than the original strain (from 114.5 to 332.1%).

In addition, the further study of these mutations can provide information for rational design at the molecular level to promote GOX production [67]. The directed evolution of GOX resulted in a double mutant (Thr30Ser Ile94Val) with improved pH stability, thermal resistance (from 58 °C to 62 °C), and k_cat_ for glucose (from 69.5/s to 137.7/s) [68]. It has been demonstrated that site-directed mutagenesis can be used to improve GOX production. The oxidase activity and dehydrogenase activity of GOX produced by the Ser114Ala/Phe355Leu mutant were reported to be as much as 2.8-fold higher than that from the wild-type one [69]. In addition, combining random with rational mutagenesis is also a useful way to enhance the thermal stability and catalytic efficiency of GOX from *Aspergillus niger* [70]. Through a combination of random mutation and rational design, the mutant (Gln90Arg/Tyr509Glu) introduced a new salt bridge near the interface of the dimeric protein structure when it bound to Thr554Met, making the GOX more stable [71].

Site-directed mutagenesis has the advantages of being more purposeful, faster, and more efficient, but the site-directed mutagenesis of targeted residues is often labor-intensive and time-consuming, and the effect of mutations on protein function is always unpredictable [72]. However, with the development of advanced computer technology, the design of enzymes’ functional properties has become reality, significantly reducing the time and effort required to achieve high yield, stability, and resistance in GOX. A highly heat-resistant GOX mutant was successfully constructed using computational design and experimental validation, and the combination of optimal mutations increased the apparent melting temperature by 8.5 °C and significantly enhanced the thermal stability [73]. Two mutants with significantly improved thermal stability, Ser100Ala and Asp408Trp, were obtained by computer-aided molecular design [74]. Predictive simulation of the introduction of disulfide bonds to GOX had been proven to be an effective strategy for improving the thermal stability of the enzyme in silico bioengineering [75].

### 4.3. Heterologous Expression of Glucose Oxidase

Heterologous expression is an important molecular technique for the expression of foreign gene proteins by bacteria, yeast, animal cells, and plant cells to enhance production efficiency. The selection of an appropriate expression system is key to producing highly efficient biologics. GOX has been used extensively in industry since the 1950s. The main strains of industrial production GOX are *Aspergillus* and *Penicillium* [25,76], and these have been used since the 1950s [77]. However, there are still some problems in the production of GOX using wild strains, such as the production of by-products (catalase, cellulase, amylase), low production efficiency, and their difficult separation and purification. Heterologous expression as an efficient solution can solve the above problems. To address these issues, researchers have worked not only on the enhancement of fungal GOX production, but also on the recombinant expression systems of microorganisms. The heterologous expression studies of GOX are shown in Table 2.

#### 4.3.1. Heterologous Expression of Glucose Oxidase by Yeast

In general, GOX are commonly expressed using yeast via periplasmic expression. The yeast expression system is a powerful tool for protein expression and analysis, which is especially suitable for the stable expression of functional foreign proteins, primarily due to its post transcriptional processing modification function. The most common tractable yeast hosts for the expression of heterologous protein are *Pichia pastoris*, *Saccharomyces cerevisiae, Hansenula polymorpha*, *Yarrowia lipolytica*, and *Kluyveromyces lactis* [78]. At present, GOX genes from a variety of sources have been successfully cloned and expressed in *Saccharomyces cerevisiae* [79] and *Pichia pastoris* [80]. *Saccharomyces cerevisiae* is an earlier microorganism that has been recognized and utilized by humans, but the commercial process of *Saccharomyces cerevisiae* was limited by the glycosylation of proteins, low protein yield, and plasmid instability [81]. Research showed that GOX was one of the largest foreign proteins that could be heterologously expressed in *Saccharomyces cerevisiae* [82], but the expression system produced over-glycosylated GOX, which may affect the catalytic performance of the enzyme [83]. *Pichia pastoris* has attracted attention as an excellent expression host due to its powerful methanol-regulated alcohol oxidase promoter (P_AOX1_), easy genetic manipulation, highly efficient secretion mechanism, and the ability to perform higher eukaryotic protein modifications [81,84]. In the *Pichia pastoris* expression system, the purification of recombinant proteins is easily conducted due to its limited yield of endogenous secreted proteins. *Pichia pastoris* can grow to very high cell densities with minimal media, and maintain the genetic stability of recombinant elements during continuous and large-scale fermentation [85]. Thus, it is a perfect candidate for industrial GOX production. Two early studies on heterologous expression of GOX in *Pichia pastoris* GS115 [76] and *Pichia pastoris* X33 [86] from *Aspergillums niger* ATCC 9029 resulted in GOX with a slightly different molecular weight and characterization (Table 2). It was confirmed that the higher molecular weight resulted from the glycosylation by the *Pichia pastoris* X-33 host machinery during the secretion process [86]. The discrepancy of characterization may be caused by the host, however, there were no later studies explaining this. *Yarrowia lipolytica* is a non-conventional yeast host with promising potential for the heterologous expression of functional proteins. The GOX gene of *Aspergillus niger,* expressed in *Yarrowia lipolytica* under the action of a strong hp4d promoter, displayed a pure and active form, and the expected molecular weight of GOX [87]. *Kluyveromyces marxianus* is a fast-growing organism with a stronger tolerance to high temperatures and wider substrate range. It is a suitable host for the production of glycosylated heterologous proteins [78].

In addition to selecting an appropriate host, many strategies have been proposed to improve the heterologous expression of GOX, such as codon optimization, increasing gene copy number, and inserting strong promoters and appropriate signal peptides into the expression vector [88]. The optimization of codons in the GOX gene, and adjustments to the C + G content according to the preference for the *Pichia pastoris,* resulted in the GOX yield accounting for 80% of the total secreted protein [89]. GOX production reached 21.81 g/L by manipulating genes involved in the protein-folding mechanism and abnormal folding stress response of genes [90]. Four different promoters (bidirectional galactose dehydrogenase 1 and 10 (GAL1, GAL10) promoters, glyceraldehyde-3-phosphate dehydrogenase (GPD) promoter, a yeast hybrid ADH2-GPD promoter consisting of alcohol dehydrogenase II (ADH2) and GPD promoter) were used to express GOX in *Saccharomyces cerevisiae*; the GADH2-GPD hybrid promoter was shown to be the best promoter for improving GOX production [82].

#### 4.3.2. Heterologous Expression of Glucose Oxidase by *E. coli*

*E. coli* is one of the most common hosts of heterologous gene expression, with certain advantages. First, *E. coli* grows very fast, with a doubling time about 20 min, and can easily reach high cell densities, which are estimated to be about 200 g in dry cell weight/L or roughly 1 × 10^13^ viable bacteria/mL [91]. Second, *E. coli* can be grown in a medium (such as LB) with inexpensive components [91]. Third, the exogenous DNA can be introduced into the *E. coli* by a simple and rapid transformation method. However, the use of *E. coli* as a heterologous expression host still has some limitations. First, protein expression in the *E. coli* system is prone to protein non-expression and low soluble protein expression. In addition, the culture of *E. coli* can form inactive inclusion bodies caused by disulfide bonding, fast production rates, and interactions with chaperones [92]. Further, posttranslational modification mechanisms such as glycosylation, phosphorylation, and N and O-terminal modifications are not available in *E. coli*. The GOX gene derived from *Penicillium amagasakiense* was transferred into *E. coli* for expression, resulting in the formation of inclusion bodies and inactive GOX. Although active enzyme was obtained after denaturation and renaturation, the enzyme was lost [93]. Inclusion bodies are usually solubilized using high concentrations of urea or guanidine hydrochloride, followed by the slow removal of the denaturant in the presence of an oxidizing agent to refold the solubilized protein [94].

Notably, GOX always presented as an inclusion body in the *E. coli* expression system. To improve the expression of target genes in *E. coli*, several different strategies have been proposed, including a change in vector, culture conditions, and the gene sequence, as well as the co-expression of molecular chaperones [95]. The incubation of *E. coli* at 6–10 °C has been reported as an effective way to overcome the problem of the insolubility of recombinant proteins (mannanase and cellulase) [96]. Molecular chaperones can promote protein expression and solubility, and a study reported that DnaK or GroEL (two major chaperones) can promote the soluble expression of mouse prion proteins in *E. coli,* which are normally insoluble [97]. Recombinant proteins without signal peptide sequences do not alter their biochemical properties and functions [95]. Therefore, this feature can be utilized to achieve high soluble expression. In addition, there are several solubility-enhancing fusion tags for *E. coli* expression, such as maltose-binding protein (MBP), glutathione-S-transferase (GST), thioredoxin (Trx), and small ubiquitin-modifier (SUMO) [98]. By taking these strategies further, the expression of GOX in *E. coli* can become a reality.

#### 4.3.3. Heterologous Expression of GOX Using Other Technologies

Cell-surface display technology is a useful recombinant technique for expressing functional proteins on the surface of microorganisms, which is achieved through the co-translational fusion of an anchor protein with a functional protein so that the latter is eventually secreted and anchored on the microbial surface [99,100]. GOX was successfully displayed on the surface of yeast cells using α-agglutinin as an anchor motif and exhibited excellent enzymatic properties, such as good stability over a wide pH range (pH 3.5–11.5), and good thermal stability [101]. The main advantage of the surface display technology is that functional proteins are displayed on the cell surface, proteases do not require complex preparation processes, and it can provide an economical alternative to traditional purified enzyme production [102]. In addition, the development of this technology is important for promoting the electrochemical application of GOX.

Cell-free protein synthesis uses crude cell extracts rather than intact cells to initiate protein synthesis [103]. While it has previously been used as a basic research tool for understanding transcription and translation, recent advances have made the large-scale synthesis of complex proteins possible [104]. An early study of GOX expression in a coupled cell-free transcription–translation system based on tobacco BY-2 cell lysates may provide an example for cell-free GOX synthesis [105]. However, there were no later studies that used this strategy for GOX production.

**Table 2:** Heterologous expression of glucose oxidase.

Source	Host	Vector	Molecular Weight (kDa)	Characterization	Activity (U/mL)	Reference
*Aspergillus niger* A9	*Pichia* *pastoris* GS115	pPIC9	75	pH 6.0, temperature = 40 °C K_m_ = 21.06 mM V_max_ = 359 μmol/min/mg	99	[3]
*Aspergillus niger* ATCC 9202	*Yarrowia lipolytica*	pINA1296	80	pH 5.5, temperature = 37 °C	0.37	[87]
*Aspergillus niger*	*Saccharomyces* *cerevisiae*	pGal	78–105	pH 6.0 specific activity 194 U/mg	10	[106]
*Aspergillus niger* NRRL-3	*Trichoderma reesei*	pRS424	70–90	NA	NA	[107]
*Aspergillus niger* Z-25	*Pichia* *Pastoris* SMD1168	pPICZαA	94	pH 6.0, temperature = 40 °C K_m_ = 16.95 mM K_cat_ = 484.26 s^−1^ specific activity 153.46 U/mg	40	[108]
*Aspergillus niger*	*Hansenula* *polymorpha*	YEpl3	71	NA	NA	[109]
*Aspergillums niger* ATCC 9029	*Pichia pastoris* GS115	pPIC9	69	pH 7, temperature = 50 °C	NA	[76]
*Aspergillums niger* ATCC 9029	*Pichia pastoris* X33	pGAPZαC	78	pH 6, temperature = 37 °C	NA	[86]
*Aspergillums niger*	*Penicillium nalgiovense*	pSK + 3.Sma	NA	specific activity 2.8 U/mL	NA	[110]
*Aspergillus heteromophus* CBS 117.55	*Pichia pastoris* GS115	pPIC9k	75	pH 6.0, temperature = 35 °C K_m_ = 187 mM V_max_ = 185.6 µmol/min/mg	NA	[84]
*Penicillium amagasakiense* ATCC 332245	*Pichia pastoris* GS115	pPICZαA	72	pH 6.0, temperature = 50 °C K_m_ = 18.2 ± 1.2 mM specific activity 365 U/mg	4	[83]
*Penicillium notatum* F4	*Pichia pastoris* GS115	pPIC9	72–95	pH 6.2, temperature = 35 °C K_m_ = 83 mM V_max_ = 2170 µmol/min/mg	148	[89]
*Penicillium amagasakiense* ATCC 28686	*Escherichia coli*	pCYTEXP1	60	pH 5.2–6.2, temperature = 28–40 °C K_m_ = 5.2–6.2 mM	NA	[93]
*Penicillium sp* MX3343	*Pichia pastoris* X33	pPICZαA	NA	pH 5.5, temperature = 30 °C K_m_ = 65.7 mM	458.6	[111]
*Cladosporium neopsychrotolerans* SL16	*Pichia pastoris* GS115	pPIC9	68	pH 7.0, temperature = 20 °C K_m_ = 103 mM	2.9	[112]

NOTE: NA, not available. Molecular weight refers a single subunit. pH: potential of hydrogen. PI: isoelectric point. Temperature and pH are the optimum conditions for application of GOX. Molecular weight, characterization, and activity column present the reported data of GOX by heterologous expression.

## 5. Conclusions

In this paper, we discuss the predictive structures of GOX from *Citrobacter freundii* and *Heliothis viriplaca,* with low similarities in their amino-acid sequences compared with GOX from *Aspergillus niger*. We also summarize the GOX inhibitors in terms of how they impact GOX activity. We mainly describe strategies for improving production. Although there has been more than 90 years of research on GOX, *Aspergillus* and *Penicillium* are the main microorganisms used in the industrial production of GOX, and many questions remain unresolved. First, it is worth finding new strains through selection in nature or metabolic engineering that can produce GOX at a low cost and with high yield, with high biocatalytic activity and stability. Another remaining challenge is understanding the structural properties of GOX, which is a major constraint on their large-scale application. With technological advances, it is believed that GOX will have greater commercial applications in the future.

## Figures and Tables

**Table 1:** The reported strains for glucose oxidase production, and its molecular weight and characterization.

Species	Genus Name	Molecular Weight (kDa)	Characterization	Source	References
*Aspergillus*	*A. niger*	75	K_m_ = 23.7 ± 0.3 mM PI = 3.7	Corn cobs	[5]
	*A. flavus*	NA	NA	Soil samples	[6]
	*A. tubingensis*	60 (three subunits)	pH 4.5 temperature = 60 °C specific activity 3435 U/mg	Contaminated cereal sample	[7]
	*A.* *terreus*	NA	NA	Soil sample	[8]
*Penicillium*	*P chrysogenum*	76	PI = 5.4	NA	[9]
	*P. funiculosum*	70	pH 6.0–8.6 PI = 4.40–4.55 specific activity 3730 U/mg	NA	[10]
	*P. pinophilum*	77.7	K_m_ = 6.2 mM specific activity 113.5 U/mg	Soil sample	[11]
	*P. adametzii*	NA	specific activity 75.8 U/mg	NA	[12]
	*P. expansum*	72	temperature = 60 °C specific activity 178 U/mg	NA	[13]
Insect	Honeybee	70	NA	Apiary of institute	[14]
	*Spodoptera exigua*	70	NA	Insect rearing facility	[15]
	*Helicoverpa* *armigera* larvae	67	NA	Cotton fields	[16]
	Locust cuticle	NA	pH 6.5 K_m_ = 28 mM	Locusts	[17]
Other	*Pleurotus ostreatus*	70 (four subunits)	pH 5.5–6.0 temperature = 50 °C K_m_ = 1.34 mM	NA	[18]
	*Goffeauzyma gastrica*	NA	NA	Soil samples	[19]
	*Talaromyces flavus*	71	pH 5.6 temperature = 37 °C K_m_ = 1.6 mM	NA	[20]
	*Aureobasidium pullulans*	NA	specific activity 1766.1 U/mg	The leaf of the mangrove plant	[21]
	*Botrytis cinerea*	35 (four subunits)	pH 7.5 PI = 4.2	Vine (*Vitis vinifera)*	[22]
	*Aureobasidium sp*	65.1	PI = 4.9	A mangrove ecosystem	[23]

NOTE: NA indicates that it is not mentioned. Molecular weight refers a single subunit. pH: potential of hydrogen. PI: isoelectric point. Temperature and pH are the optimum conditions for GOX’s application.

## Data Availability

Not applicable.
